# Fungal Pigments: Carotenoids, Riboflavin, and Polyketides with Diverse Applications

**DOI:** 10.3390/jof9040454

**Published:** 2023-04-07

**Authors:** Maria Afroz Toma, Md. Hasibur Rahman, Md. Saydar Rahman, Mohammad Arif, K. H. M. Nazmul Hussain Nazir, Laurent Dufossé

**Affiliations:** 1 Department of Food Technology & Rural Industries, Bangladesh Agricultural University, Mymensingh 2202, Bangladesh; hasibur46782@bau.edu.bd (M.H.R.); saydurr182@gmail.com (M.S.R.); 2 Department of Microbiology and Hygiene, Bangladesh Agricultural University, Mymensingh 2202, Bangladesh; mdarif38515@bau.edu.bd (M.A.); nazir@bau.edu.bd (K.H.M.N.H.N.); 3 Laboratoire de Chimie et de Biotechnologie des Produits Naturals, CHEMBIOPRO EA 2212, Université de La Réunion, ESIROI Agroalimentaire, 97744 Saint-Denis, France; 4 Laboratoire ANTiOX, Université de Bretagne Occidentale, Campus de Créac’h Gwen, 29000 Quimper, France

**Keywords:** natural pigments, carotenoids, riboflavin, polyketides, fungi, colorants

## Abstract

Natural pigments and colorants have seen a substantial increase in use over the last few decades due to their eco-friendly and safe properties. Currently, customer preferences for more natural products are driving the substitution of natural pigments for synthetic colorants. Filamentous fungi, particularly ascomycetous fungi (*Monascus*, *Fusarium*, *Penicillium*, and *Aspergillus*), have been shown to produce secondary metabolites containing a wide variety of pigments, including β-carotene, melanins, azaphilones, quinones, flavins, ankaflavin, monascin, anthraquinone, and naphthoquinone. These pigments produce a variety of colors and tints, including yellow, orange, red, green, purple, brown, and blue. Additionally, these pigments have a broad spectrum of pharmacological activities, including immunomodulatory, anticancer, antioxidant, antibacterial, and antiproliferative activities. This review provides an in-depth overview of fungi gathered from diverse sources and lists several probable fungi capable of producing a variety of color hues. The second section discusses how to classify coloring compounds according to their chemical structure, characteristics, biosynthetic processes, application, and present state. Once again, we investigate the possibility of employing fungal polyketide pigments as food coloring, as well as the toxicity and carcinogenicity of particular pigments. This review explores how advanced technologies such as metabolic engineering and nanotechnology can be employed to overcome obstacles associated with the manufacture of mycotoxin-free, food-grade fungal pigments.

## 1. Introduction

Pigments, also known as dyes in some applications, have a wide range of uses in our daily lives. Color has a variety of connotations, ranging from traffic signals to the fitness of edible goods for ingestion (fresh, ripe, safe, nutritional, or rotting), as well as arts and clothes [1,2]. The present global pigment consumption is over 9.7 million tons, and the demand for natural pigment was expected to reach nearly 10 million tons by 2017 [3]. The global market for food colorants was estimated to be USD 3.88 billion in 2018, and it is anticipated to reach USD 5.12 billion by 2023 with a compound annual growth rate (CAGR) of 5.7% [4]. For example, the worldwide demand for carotenoids (astaxanthin, beta-carotene, canthaxanthin, lutein, lycopene, and zeaxanthin) was expected to be USD 1.53 billion by 2021 [4], and it is growing every day. As plant-derived carotenoids are expensive, scientists have become increasingly interested in bacterial carotenoid production in recent years due to its sustainability and cost-effectiveness [5].

Pigments or colorants have always fascinated people, and they can be either natural or synthetic. By virtue of the various carcinogenic and teratogenic effects of synthetic dyes [6], humans have always preferred natural pigments [7,8], such as those found in plants, animals, microorganisms, and insects [7,9]. Among all natural sources, plants are a good supply of pigments, although their production may not be under control due to several difficulties such as seasonal dependency, geographic factors, and variations in color intensity and hues [10]. Therefore, analysts and industries incline toward microorganisms as a result of the practicality of creation [11] and the extraction of colorants [12]. Additionally, different factors are also considered, such as simple development, the opportunity for consistent collection, flexibility under different conditions with no or few incidental effects, eco-accommodating nature, biodegradability, having a higher water dissolvability than plant colorants, and the most compelling: minimal expenses [13]. Likewise, the microbial colorant has some unavoidable applications in multidisciplinary viewpoints; for instance, in environmental, agribusiness, biomedical, and mechanical investigations [14,15].

Microorganisms, unlike higher organisms, are a source of easily renewable resources that can lead to higher yields [16]; among these, natural pigments have attracted industry’s attention due to a growing interest in the development of new, safe, easily degradable, environmentally friendly products with no adverse effects [17]. This focus on microbial pigment may be attributed to the large-scale use of artificial pigments and dyes in the production and manufacture of foods, dyes, cosmetics, and medications, all of which have had a variety of negative consequences [18]. Natural pigments have a significant part in the physiology and molecular processes of microorganisms because they serve as a means of adaptation to varied severe conditions, provide protection from solar radiation, and take part in functional processes such as photosynthesis [19].

Microbial pigments can be obtained from diverse sources, including bacteria, yeast, fungi, parasites, basidiomycetes, and microalgae, which have a variety of applications in food, pharmaceuticals, colorants, and imaging [7,20]. Among many microorganisms, fungi are of interest because of their speedy growth patterns, which can also be genetically controlled to promote larger pigment production [21]. In comparative studies of microorganisms, pigments from growths have also drawn increasing attention due to their easy maintenance in terms of high light, heat, and an unfriendly pH, their wide range of shadings, their compound consistency, and their dissolvability in water [22]. Fungal systems that are inexpensive and easily controlled can be used for pigment purification and extraction on a wide scale owing to the developments in fermenters and downstream processing [23,24]. Any harmful metabolite cannot enter the primary product through the bio-pigment purification process [4].

Carotenoids, riboflavin, lycopene, melanins, quinones, and betalains are the most common and widely used biopigments obtained from fungi. *Monascus*, *Cordyceps*, *Serratia*, *Penicillium*, *Aspergillus*, *Fusarium*, *Talaromyces*, and a variety of other fungi are capable of producing such pigments [25]. Monascorubramine, a red pigment derived from *Monascus* sp., has traditionally been used in the production of East Asian red rice [26] and red bean curd [27]. In addition to its shading properties, the fungal colorant has some significant qualities such as antimicrobial [28], antioxidant [12], anticancer, and cytotoxic activities [29], which have extended its utilization in various areas [30]. Microbial pigments are thus more than just coloring chemicals because of their allied biological functions, which may be advantageous to humans. This review endeavors to bring together the up-to-date issues and details of fungal colorants and their significant beneficial properties in order to relate them to potential biomedical and human health applications.

## 2. Pigment-Producing Fungi in Nature

Although pigments are produced by plants, animals, and microorganisms, fungi are the main source from which pigments are being isolated [31]. Fungi that produce pigments can be obtained from different sources, such as soil [32], mangroves, and aquatic environment, as clarified by Kalra and his research groups [33]. The ocean biological system, the mangrove environment, and the earthly biological system are, on the whole, wellsprings of normal or natural colorants that produce filamentous growths.

Fungi that can be found in marine living spaces foster auxiliary metabolites with greater variety, supporting their endurance in an extremophilic environment and bringing about the formation of a few remarkable mixtures known as pigments [34,35]. *Microsporum* sp. and *Penicillium bilaii* are both marine fungi isolated from the Huon estuary, and they produce yellow-colored fungal polyketide pigments (citromycetin and citromycin) [36]. Additionally, *Microsporum* sp. also produce a similar yellow compound known as flavoglaucin [37]. Researchers discovered *Talaromyces* sp. and *Trichoderma atroviride* strains in maritime sediment as possible producers of red pigments known as azaphilones [14]. Extracellular metabolites produced by halophilic fungi are important in biotechnological applications since they are abundant [38]. Melanin is a pigmented molecule in this family that plays a significant role in a variety of cosmetic and medicinal uses. *Trimmatostroma salinum* and *Phaeotheca triangularis*, halophilic marine fungal strains found along the eastern coast of the Adriatic Sea, produce melanin colors in saturated sodium chloride solutions [39].

In a specific ecosystem, mangrove ecosystems have a surprisingly diversified habitat of both marine and terrestrial environments. Despite their high potential for producing a diverse range of natural pigments, only a few mangrove fungi have been researched thus far [40]. Approximately 100 mangrove fungi were isolated from India’s Godavari mangroves, with the majority of the strains being pigment makers [41]. These isolates may generate a wide range of colors, including green, gray, black, brown, orange, yellow, purple, and violet [42]. Another endophytic fungus, *Alternaria* sp., which is isolated from mangrove tree fruit, was able to generate yellow to red polyketides known as anthraquinones. However, altersolanol A, alterporriols C–M, macrosporin, dactylariol, tetrahydroaltersolanol B, and physcion were discovered as polyketide compounds [43].

Filamentous fungi that generate different colorants are common in terrestrial environments. In research, four bianthraquinone and two monoanthraquinone compounds with orange-red colors were discovered in a soil specimen of volcanic ash from Japan [44]. Fusarium verticillioides was discovered to be a possible generator of naphthoquinone pigment in soil from Chiang Mai, Thailand [45], while *P. sclerotiorum* 2AV2 was reported to generate highly colored pigments [32]. Two terrestrial strains, *Penicillium purpurogenum* and *Fusarium oxysporum*, were discovered as promising makers of red pigment [25]. In a recent study, a fungal strain of *Penicillium* sp. (GBPI P155) isolated from high-altitude soil in the Indian Himalayan area was shown to generate a dark orange color and have actinobacterial activity [46].

## 3. Natural Colorants from Different Fungi

Fungal pigments are produced as secondary metabolites when essential nutrients in the culture medium are depleted or the environment is unfavorable for growth [47,48] *Monascus*, *Aspergillus*, *Penicillium*, *Fusarium*, *Trichoderma*, *Talaromyces*, etc., are some examples of fungi that produce pigments as secondary metabolites [49]. Various colors are produced by the fungi, viz., red [50], yellow [51], and orange [52] colors by *Monascus*, yellow and brown colors by *Aspergillus*, a blue color by *Lactarius* [53], a pink/violet color by *Fusarium* [54], and so on.

**Figure 1:**
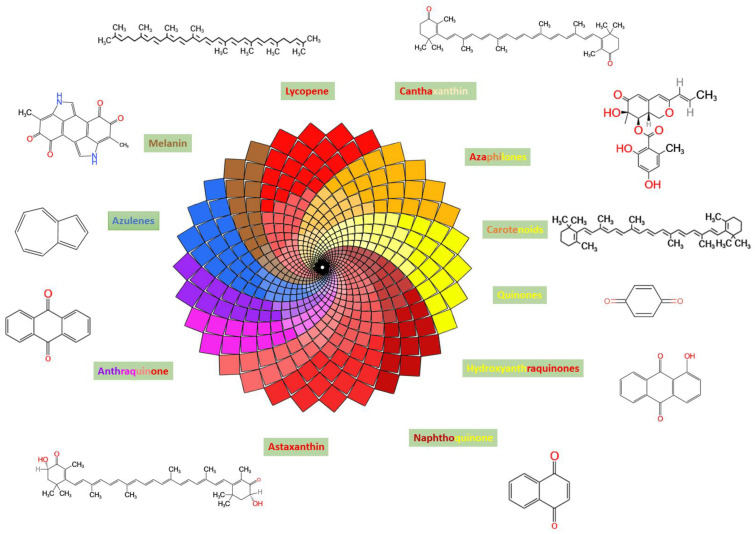
Fungal pigments (carotenoids and polyketides) exhibiting their color and their typical structure skeletons.

Figure 1 depicts some of the shades and hues of colors produced by filamentous fungi as a result of several classes of secondary metabolites and their fundamental chemical structures. The first commercial red color, produced from the fungal strain *Penicillium oxalicum* var. *armeniaca* CCM 8242, which was isolated from soil, was Arpink redTM pigment [55]. Many of these colors are produced by ascomycetous fungi and are mostly polyketide derivatives. Table 1 shows some of the common pigments generated by these fungal species to present a general idea of the range of colors and hues they may create.

*Monascus* sp., a pigment-producing filamentous fungus that belongs to Ascomycetes and the Monascaceae family, is an essential fungus for the manufacture of industrial pigments and is used extensively as food coloring [88]. It has been revealed that there are four different species of *Monascus* that may generate pigment, including *Monascus purpureus*, *M. froridanus*, and *M. pilosus* [89]. There are six primary types of pigments produced by the *Monascus* species: polyketide pigments that are yellow, orange, and red in hue [90]. *Monascus purpureus* (ATCC16436) may generate orange and red pigments during solid-state fermentation using maize cob and glycerol [91]. Monascorubramine and rubropuntamine are red-colored polyketides, while monascin and ankaflavin are yellow-colored polyketides, and monascorubrin and rubropunctatin are orange-colored polyketides [29]. *Monascus* pigments are found to be more stable in solution at a neutral or alkaline pH [92], and the yellow pigments are more stable in solution than red and orange pigments [93]. Another study discovered that *Monascus ruber* SRZ112, a new endotype, can produce natural colors from agro-industrial wastes [94].

Based on a chemotaxonomic examination, the polyketide pigments generated by Ascomycetous fungi have been deemed safe [95]. The study primarily emphasized species from two major genera (*Penicillium* and *Epicoccum*) as possible pigment producers for future pigment production. *P. purpurogenum*, *P. aculeatum*, *P. funiculosum*, and *P. pinophilum* are among the *Penicillium* strains that may generate azaphilones and amino acid derivatives [95]. These strains do not generate the mycotoxin citrinin or any other mycotoxin that is harmful to humans in their polyketide pathway [79]. However, four of these strains can release extracellular colors into the liquid medium, indicating that they might be used as cell factories in the future. Many species of *Penicillium* are safe for humans. However, some, such as *Penicillium crateriforum*, *Penicillium islandicum*, *Penicillium rugulosum*, *Penicillium variabile*, and *Penicillium marneffei,* are not [95]. In one research study, an unnamed species of *Penicillium* was found to generate pigments, suggesting that it has the potential to function as both a food colorant and a nutraceutical due to its radical-scavenging characteristics [96]. Another study examined the effects of temperature variations on the generation of natural colors in the *Penicillium* sp. (GEU 37), a strain of fungus that can withstand cold and acidic environments. In potato dextrose, the fungus produces more sporulation, exudate, and red pigment at 15 °C than it does at 25 °C. In addition to various other significant bioactive chemicals, LC-MS analysis found the existence of carotenoid derivatives, derivatives of chromenone, and derivatives of hydroxyquinoline as key compounds [97]. *Penicillium hirayamae* U., a known producer of azaphilone that has received little attention chemically, was examined by a molecular networking study [98], which resulted in the isolation of three new azaphilones and penazaphilones J-L. *Penicillium purpurogenum* is a promising fungal strain for the production of natural pigments that may provide a practical, ecologically friendly alternative to the current sources of colors for use in the future food business and textile industry [99].

*Aspergillus* species may generate a variety of colors, including black by *Aspergillus niger* [58], brown by *Aspergillus niger* [57], red by *Aspergillus flavus* [59], and a yellow color by *Aspergillus sclerotiorum* and *Aspergillus versicolor* [60]. After drying, *Aspergillus glaucus* produced a variety of pigments, including auroglaucin (orange-red colors), flavoglaucin (lemon-yellow hues), and rubroglaucin (ruby-red hues) [100].

*T. purpurogenus* and allied species, such as *Talaromyces amestolkiae*, *Talaromyces ruber*, and *Talaromyces stollii*, have been found to generate pigments that are non-mycotoxigenic and non-pathogenic to humans [86]; however, their individual mycotoxin profiles and pigment-generating capacities have yet to be investigated. *T. purpurogenus* strains have recently been studied for their potential pigment synthesis and their advantages over water-soluble, extracellular *Monascus*-like pigments [24].

In a recent study, riboflavin and chlioquinol II, two components, were found in the yellow pigment producer *Arcopilus aureus*. With respect to other natural dyes, the pigment remained relatively stable when heated [101]. The red pigment produced by *Saccharomyces cerevisiae* mutants is a newly discovered pigment that is currently being explored. It is made up of 1-(5′-phosphoribosyl)-5-aminoimidazole containing amino acid residues and has a molecular weight range of 2 to 10 kDa [102].

## 4. Fungal Carotenoids, Riboflavin and Polyketides

Carotenoids and polyketides are among some of the natural colors that fungi produce [103]. In contrast to carotenoids, which are constructed of terpenoids that have 40 carbons in their main chain, fungal polyketides are composed of octaetides and tetraetides, which form 8 C2 units to construct the polyketide chain [104,105]. Carotenoids, for example, are made up of molecules such as β–carotene, astaxanthin, and lycopene (Figure 1), whereas polyketides are made up of molecules such as melanins, anthraquinones, hydroxyanthraquinones, azaphilones, oxopolyene, quinones, and naphthoquinone (Figure 1) [106,107,108].

### 4.1. Fungal Carotenoids

Carotenoids are terpenoids with 40 carbons. They are organic substances. They are natural compounds that vary from yellow to orange-red in color and can safeguard against photo-oxidation [102,104]. It is believed that exposure to light, particularly blue light, is the main trigger for carotenogenesis [109]. Light-absorbing conjugated double bonds, which are mostly responsible for the physiochemical characteristics of carotenoids, are considered the most significant structural feature of these pigments. For example, the hue of most carotenoids is a result of conjugated double bonds. A colorful carotenoid can only be obtained with the help of at least seven conjugated double bonds [102,110,111]. Two categories can be used to categorize carotenoids. “Oxygen-free carotenes”, such as β-carotene, lycopene, and torulene, are one of them. “Oxygen-containing xanthophylls”, such as astaxanthin, canthaxanthin, and torularhodin, make up the second category [112]. Food colorants such as carotenoids are frequently being utilized in the food sector because of their powerful antioxidant effects.

#### 4.1.1. β–Carotene

**Figure 2:**
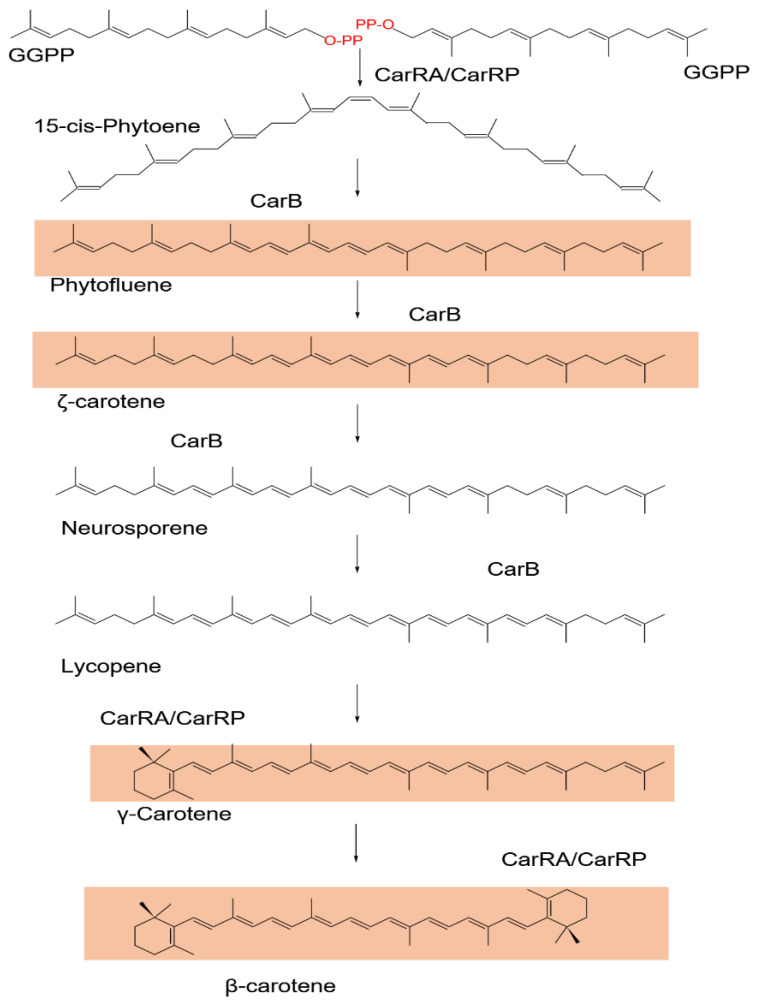
Biosynthetic pathway of carotenoid (from GGPP) from fungi. The figure has been reproduced following Avalos [120,121].

One of the most significant carotenoids is β-carotene. β-carotene, commonly known as pro-vitamin A, is a yellowish carotenoid pigment with antioxidant and disease-fighting potential [113,114]. *P. blakesleeanus*, *M. circinelloides* [115], and *B. trispora* are examples of mucorales fungi that contain yellowish-carotene, one of nature’s most abundant carotenes. The filamentous fungi *Sclerotium rolfsii* and *Sclerotinia sclerotiorum* and the ascomycetes *Aspergillus giganteus*, *Cercospora nicotianae*, and *Penicillium* sp. have all been reported to produce β-carotene [116]. Two industries in Russia and Spain have recently produced β–carotene from *B. trispora*, and the sexual stimulation of carotene biosynthesis is required to enhance pigment yields up to 35 mg/g [117]. Food and raw materials examined by the European Union Committee can utilize the beta-carotene generated by *Blakeslea trispora* fermentation as a coloring agent. When exposed to blue light impulses, wild strains of *M. circinelloides* become activated due to substantial changes in the β–carotene structural genes and generate a high amount of yellow pigment. The essential characteristics of the carotenoid pathway (Figure 2) in *Phycomyces* and *Mucor* are identical, including photocarotenogenesis [118]. These genes are significantly activated in the *Mucor circinelloides* when exposed to blue light. This results in high pigment concentrations [119]. The most recent method for producing β-carotene industrially has been to use metabolic engineering to build microbial cell factories. Yeasts such as *Saccharomyces cerevisiae* and *Yarrowia lipolytica* have garnered the most interest due to their robustness for large-scale fermentation, security, and sophisticated genetic manipulation tools. Carotenoids that use the native mevalonate pathway also get an advantage [114].

#### 4.1.2. Lycopene

In addition to its red color, lycopene has the longest beta-carotene isomer of any carotenoid, with an open-chain, unsaturated carotenoid molecule. It is also known as psi-carotene, and it is insoluble in water and is extremely sensitive to heat and oxidation. Due to the number of double bonds in its structure, lycopene has about 1056 possible isomers, yet only a handful are observed in nature. Using maize fiber material as a substrate, a genetically engineered mold, *Fusarium sporotrichioides*, was utilized to produce colorants and the antioxidant lycopene [68]. The highest in vitro lycopene production (0.5 mg/g of dry mass) was observed in five- to six-day-old cultures [122]. *Talaromyces amestolkiae* is a natural producer of red colorants in both the extracellular and cell-bond state; therefore, it is very important for the dye industry [123].

#### 4.1.3. Canthaxanthin

Canthaxanthins may be a good source of antioxidants that can inhibit lipid oxidation. *Chlorociboria aeruginosa’s* quinone xylindein gives its wood a green hue [124]. *Tricholoma aurantium*, similar to orange-red cups, has a pigment called aurantricholone in which a pyragallole ring is linked to pulvinic acid and oxidatively dimerized, resulting in a calcium complex [125]. Despite the fact that fungi are non-photosynthetic organisms, several species, including as *Blakeslea trispora*, *Phycomyces*, and *Neurospora crassa*, have been shown to contain carotene hydrocarbons. Carotenoid pigment canthaxanthin is produced by the bacterium *Cantharellus* sp. [126].

#### 4.1.4. Astaxanthin

Among the carotenoids with antioxidant properties, astaxanthin can be highlighted due to its pharmaceutical, feed, food, cosmetic, and biotechnological applications [127]. A carotenoid pigment, astaxanthin is a 40-carbon tetrapene made up of connected isoprene molecules. It is chemically known as 3, 3′-dihydroxy-b, b-carotene-4, 4′-dione [128]. It was discovered that astaxanthin outperformed carotene and lutein in preventing degradation of lipids, including membrane phospholipids [129,130]. Compared to beta-carotene and vitamin E, it has ten and one hundred times more anti-oxidative action [131]. Microorganisms such as red basidiomycetous yeast *Xanthophyllomyces dendrorhous* generate astaxanthin, an orange-red pigment. When using *Xanthophyllomyces dendrorhous* yeast for the commercial manufacture of the pigment, a low molecular concentration of astaxanthin is a major issue [132].

#### 4.1.5. Torulene

Torulene (C_40_H_54_) is chemically known as 3′,4′-didehydro-β,γ-carotene. Depending on the concentration, it is orange-red or orange in hue [102]. It can be used as an additive in food, cosmetics, and feed and has potent anti-microbial [133] and anti-oxidative [134] qualities. Using Trolox, Dimitrova and team members evaluated the antioxidative activity (ORAC) of torulene [134]. This measure for torulene had value of 2.77, which was lower than the anti-oxidative activity found for β-carotene. Rat and mouse experiments revealed that it seems to have anti-cancerous qualities [135]. Fungi from the genera *Dioszegia*, *Rhodotorula*, *Sporidiobolus*, *Cystophilobasidium*, *Neurospora*, *Rhodosporidium*, and *Sporobolomyces* produce torulene [135].

#### 4.1.6. Torularhodin

Torularhodin (C_40_H_52_O_2_) is chemically known as 3′,4′-didehydro-β,γ-caroten-16′-oic acid. Fungi belonging to the genera *Rhodotorula*, *Sporidiobolus*, *Cystofilobasidium*, *Rhodosporidium*, and *Sporobolomyces* produce torularhodin. Like torulene, torularhodin also can be used as an additive in food, feed and cosmetics and has potent ant-microbial and anti-oxidative qualities. Among the most important producers of torularhodin are *Rhodotorula*, *Sporobolomyces*, and *Sporidiobolus* [135]. Torularhodin from *Rhodotorula glutinis* was found to be more effective than carotene at scavenging peroxyl radicals and halting the decomposition of singlet oxygen [136]. In a different research study, it was discovered that torularhodin inhibited lipid peroxidation, and its inhibitory action was better than that of α-tocopherol [137].

### 4.2. Fugal Riboflavin

Fungi and many other microbes generate riboflavin, a yellow-colored, water-soluble vitamin. Rather than using classic chemical synthesis methods, advanced biotechnological technologies are being used to synthesize riboflavin. Riboflavin is predominantly generated by three microorganisms, the ascomycetes *Ashbya gossypii* [53], the filamentous fungus *Candida famata* [138], and the bacterial species *Bacillus subtilis*, utilizing commercial competitive biotechnological techniques [139]. In terms of yield and genetic stability, the most frequently used strain is *A. gossypii* [140]. These strains are being employed to extract natural yellow colorant for a variety of food items such as fruit drinks, morning cereals, pastas, sauces, processed cheese, vitamin-enhanced milk products, and some energy beverages.

### 4.3. Fungal Polyketides

Several fungi, including the majority of filamentous ascomycete genera, produce a large number of polyketide-based pigments that have a fungal origin [141]. Fungal polyketides are composed of tetraketides and octaketides with eight C2 units that link together to create a polyketide chain. Naturally occurring polyketide pigments produced by fungi include melanins, anthraquinones, hydroxyanthraquinones, azaphilones, quinones and naphthoquinones [62].

#### 4.3.1. Melanins

Melanin is a biopigment that may be found in microbes, plants, mammals [142], cephalopoda, and sea cucumbers [143], characterized as eumelanins, pheomelanins, and allomelanins as indolic polymers [144]. Most melanins are brown or black in color, although additional hues have been identified in other studies [145]. Antimicrobial, anti-inflammatory, antioxidant and immunogenic qualities [146] are only a few of their numerous benefits, as they have the capacity to defend against environmental stress [113]. Melanin is found in all biological systems [147]. Additionally, it is produced by a variety of microbes, including *Colletotrichum lagenarium*, *Magnaporthe grisea*, *Cryptococcus neoformans*, *Paracoccidioides brasiliensis*, *Sporothrix schenckii*, *Aspergillus fumigates* [148], *Vibrio cholerae*, *Shewanella colwelliana*, *Alteromonas nigrifaciens* [149], and many species of the genus *Streptomyces* [150]. Cosmetics, photo-protective creams, and eyewear all contain melanin, which has anti-HIV effects and is helpful for photovoltage production and fluorescence research, among other things. Melanin is also utilized to produce monoclonal antibodies for the treatment of human metastatic melanoma. Fungal melanins are used as a novel biopolymer in the field of material engineering in addition to their protective function and mechanism of resistance against unfavorable conditions, which make them potential bio-compounds in food and the medicine business [151].

#### 4.3.2. Anthraquinones

The most frequent pigment class that has been shown to be possibly safe for human ingestion is anthraquinone [152]. *Aspergillus* sp., *Eurotium* sp., *Fusarium* sp., *Drechslera* sp., *Penicillium* sp., *Emericella purpurea*, *Curvularia lunata*, *Mycosphaerella rubella*, and *Microsporum* sp. are the most frequent genera that produce anthraquinones [54,74]. Researchers have indicated that several species of fungi generate anthraquinones, which have a wide range of chemical structures that may affect their ability to produce a quinoidal pigment of a certain kind [74]. According to an investigation, Rubroglaucin pigments are a composite of physcion and erythroglaucin hydroxyanthraquinones [153].

#### 4.3.3. Hydroxyanthraquinones

In nature, fungal hydroxyanthraquinoid (HAQN) pigments are found in a variety of species, including plants, insects, mammals, and microbes, including filamentous fungi of the genera *Penicillium* and *Aspergillus*. For instance, the emodin pigment can be obtained from *Penicillium citrinum* and *Penicillium islandicum* strains [154]. The food colorant Arpink redTM (now Natural RedTM) is made by a Czech business as the first commercial product within this chemical family. It is synthesized by fermentation, leveraging a soil-isolated strain of the fungus *Penicillium oxalicum* [76]. According to the findings, several *Aspergillus* sp. strains, including *A. glaucus* and *A. cristatus*, are capable of producing yellow and red HAQN. Emodin and physcion (yellow colorants), questin (yellow to orange-brown colorants), erythroglaucin (red colorant), and catenarin and rubrocristin (red colorants) are among the polyketide pigments of HAQN compounds [74,155].

#### 4.3.4. Azaphilones

**Figure 3:**
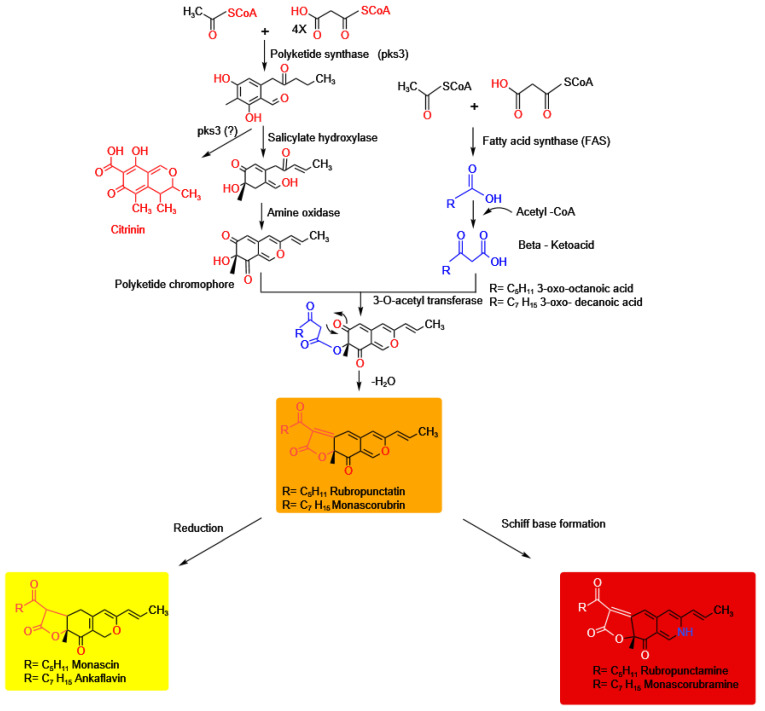
Hypothetical pathway of *Monascus* pigment and citrinin biosynthesis. The figure was reproduced following Woo and Chen [161,162].

Azaphilones are fungal polyketides pigments made by a variety of bacidiomyceteous and ascomyceteous fungi that have a highly oxygenated pyranoquinone bicyclic core [98]. Chen et al. (2020) grouped the naturally occurring azaphilones into 13 different groups: citrinins, nitrogenated, austdiols, bulgariolactones, deflevtins, spiro-azaphilones, lactone, O-substituted, hydrogenated, pulvilloric acid, chaetovirins, cohaerins, and sclerotiorins [156]. Only nine fungal genera—*Aspergillus*, *Monascus*, *Chaetomium*, *Penicillium*, *Hypoxylon*, *Muycopron*, *Phomopsis*, *Talaromyces*, and *Pleosporales*—had azaphilones had isolated from them [157]. The oldest known source of azaphilone colors is a genus of fungi called *Monuscus*, which is still regarded as one of the most prolific producers of pigments in modern times [158]. Azaphilones pigments are a category of secondary metabolites produced by *Monascus* sp. [159]. The six azaphilones that make up the main *Monascus* pigments (Figure 3) are grouped into three categories. In the first category, rubropunctatin and monascorubrin are orange pigments; rubropunctamine and monascorubramine are red pigments; and monascin and ankaflavin are yellow pigments, reduced versions of orange pigments [90]. Orange pigments are biosynthesized first, and subsequently red and yellow pigments are considered to be produced from orange pigments, depending on culture circumstances [89]. Due to its pH stability across a wide temperature range and high temperatures, *Monascus* pigments are utilized as natural food colorants. *M. purpureus* grown with ammonium chloride can create orange pigments that have antibiotic properties against bacteria, yeasts, and some filamentous fungi, according to the research [160].

#### 4.3.5. Quinones

Quinones and related conjugated compounds make up the majority of fungal pigments; however, their pigmentation in fungi can change with age [163]. Polyketide pigments produced by fungi such as *Penicillium*, *Aspergillus*, and *Helminthosporium* are known as quinones, and they are quite prevalent. The pigments auroglaucin and flavoglaucin were experimentally formulated in those species in the 1930s and 1940s [164]. *Aspergillus fumigatus* [165] produced fumigantin, which was initially yellowish-brown in color but became purple after being treated with an alkali [153].

#### 4.3.6. Naphthoquinones

When stressed, fungi release naphthoquinone pigments, just as they do with other pigment-producing species. *Cordyceps unilateralis* strain BCC 1869 is a prospective source of polyketide naphthoquinone red pigments [65], which are of particular relevance because of the chemical and structural similarities between shikonin and alkanin, two commercially available, plant-derived red pigments. The naphthoquinones were discovered as 3, 5, 8-trihydroxy-6-methoxy-2-(5-oxohexa-1,3-dienyl)-1,4-naphthoquinone, which is light-, heat-, acid-, and alkali-stable. A further potential producer of naphthoquinone pigment, *Epicoccum nigrum*, has been investigated for prospective large-scale production on rice-based media in both liquid and solid form [79].

## 5. Fungal Carotenoid and Polyketide Pathways

### 5.1. Carotenoid (β-Carotene) Biosynthesis Pathway

Carotenoids are tetraterpenoids produced from phytoene, a colorless precursor formed by the collision of two GGPP (C20 diterpene geranylgeranyl pyrophosphate) molecules colliding head-to-head (Figure 2), a process catalyzed by the enzyme phytoene synthase [120]. A chain of conjugated double bonds is generated from the phytoene hydrocarbon backbone in all carotenoid routes to absorb visible light, which is generally in the blue part of the spectrum. The chromophore provides the various carotenoids with a yellow, orange, or reddish coloring, depending on their unique absorption spectra [166]. In *Phycomyces blakesleeanus*, phytoene is produced in a cis configuration and is then isomerized to its trans isomer in the first desaturation step [167]. Without oxygen, carotenoids are known as carotenes (carotenoids without oxygen). There are, however, oxidative stages in the carotenoid biosynthesis pathways that culminate in the formation of xanthophylls [121].

Four desaturations on the phytoene backbone are required for β-carotene production, resulting in the crimson intermediate lycopene and the -cyclization of both ends of the molecule (Figure 2). In photosynthetic organisms, phytoene and ζ-carotene desaturases, two distinct enzymes, are responsible for each desaturation [168]. In fungi, however, the four desaturations are carried out by a single enzyme. In photosynthetic species, however, different genes encode the phytoene synthase and cyclase enzymes. Thus, only two fungal genes are required to produce β-carotene from GGPP, one encoding a bifunctional phytoene synthase/lycopene cyclase and the other encoding a desaturase. The desaturase gene is known as gene *carB*, and the phytoene synthase/lycopene cyclase gene, known as *carRA* or *carRP*, is connected to gene *carB* in the genome and is divergently transcribed from a shared upstream region, forming a single regulatory unit [169,170].

### 5.2. Polyketide Pathway

Tetraketides to octaketides, which contain four or eight C2 units contributing to the polyketide chain, are examples of fungal polyketide pigments. Anthraquinones, hydroxyanthraquinones, naphthoquinones, and azaphilones are examples of classes that each display a wide range of colors (Figure 1). More than one study has confirmed the increased pigment output is partly due to carbon-deprivation stress inhibiting central carbon metabolism and increasing the acetyl-CoA pool. *Monascus* pigments have a broad range of uses in the food business [171,172]. Therefore, researchers have tried to determine how they are made [173]. Five moles malonate and one mole acetate are condensed to produce a hexaketide chromophore in this biosynthetic pathway. At the same time, the fatty-acid biosynthetic pathway produces β-keto acid from a medium-chain fatty acid called octanoic acid. Monascorubrin and rubropunctatin are formed when β-keto acid and hexanoic acid transesterify (orange pigment). Ankaflavin and monascin (yellow pigment) are generated by reducing monascorubrin and rubropunctatin, respectively, while monascorubramine and rubropunctamine (Figure 3) are made by amination to obtain red pigments [159].

Experiments with radioactively tagged octanoic acid in the culture media indicated a possible biosynthetic route for the orange pigment monascorubrin, which consists of a mixture of polyketide and fatty acids [174]. The polyketide gene cluster and the route for monascorubrin biosynthesis in the filamentous fungus *Penicillium marneffei* for the manufacture of azaphilones with black, yellow, and red colors were described [161]. There are more than 16 chemical compounds in *P. marneffei’s* red pigment. These are amino acid conjugates of monascorubrin and rubropunctatin, since amino acids may be conjugated under particular circumstances without enzyme catalysis, namely via Schiff base formation (Figure 3), which is responsible for the production of ankaflavin and citrinin, a mycotoxin with nephrotoxic activity in mammals [161]. Individual amino acids are added to the growth medium to change the color of rubropunctamine and monascorubramine derivatives [175]. The genome of *P. marneffei* contains 23 putative polyketide synthase (PKS) genes and 2 putative PKS-nonribosomal peptide synthase hybrid genes [161]. The citrinin PKS C6.123 gene was also discovered in the genome [90], opening the door for research into nonmycotoxin-producing strains if the citrinin gene can be suppressed without affecting the strain’s capacity to produce colors, which appears to be possible [176]. As a result, the PKS gene responsible for the synthesis of citrinin was damaged, but the red pigment production from the fungus remained unaffected, indicating that the two routes are distinct (Figure 3). However, it remains unclear if mevinolin/lovastatin-free and citrinin-free red pigments can be made from *P. marneffei* as the latter, a mycotoxin, appears to be an early by-product of the metabolic process.

## 6. Potential Application of Fungal Pigments

Since natural pigments have benefits over synthetic pigments, their popularity has grown considerably in recent years [24]. It has been demonstrated that fungi are a reliable, accessible, alternative supply of natural pigments [12,177]. Applications for fungal pigments include food coloring, antimicrobial defense, antioxidant agents, cancer prevention, and so on. Table 1 displays fungi pigments and possible uses for them.

### 6.1. Pigments as Food Colorants

**Figure 4:**
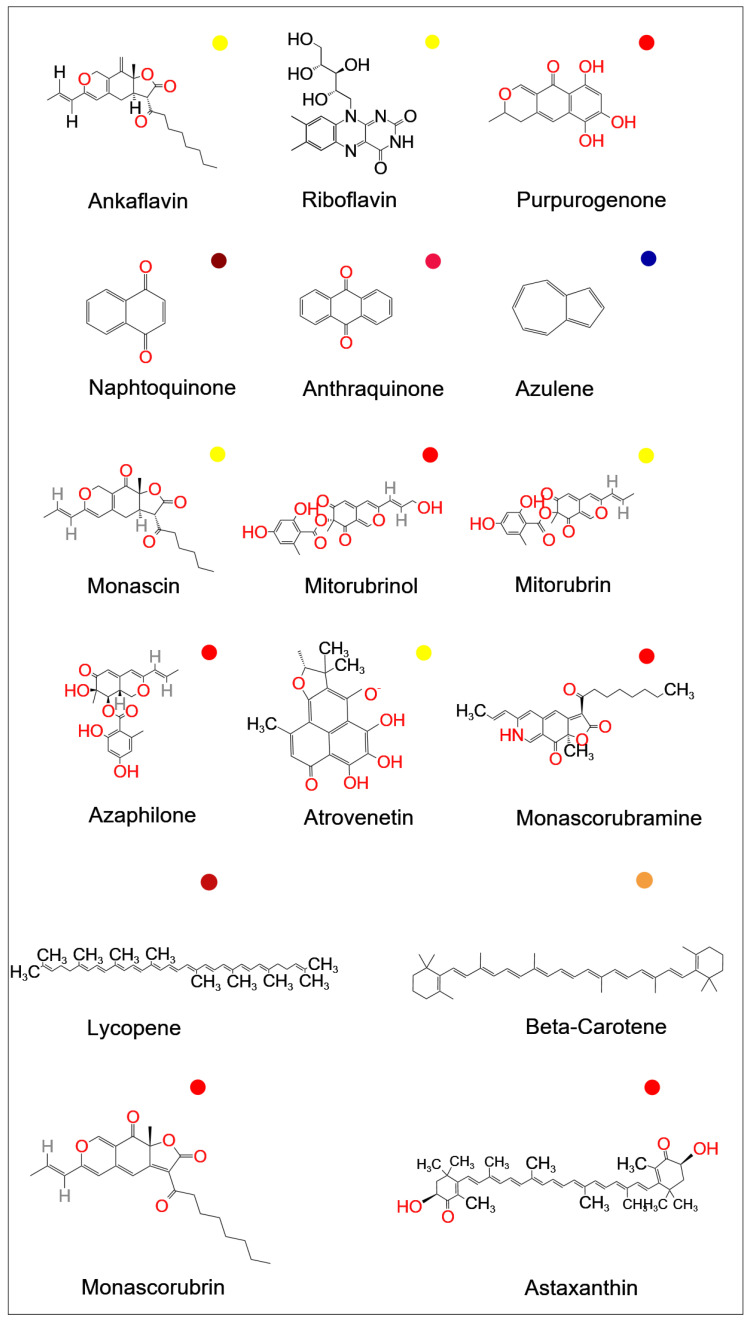
Chemical structure of some available fungal food pigments (Source: National Center for Biotechnology Information. PubChem Compound Database; (https://pubchem.ncbi.nlm.nih.gov); accessed on 1 April 2023).

The use of natural colorants enables the replacement of potentially dangerous synthetic dyes [178,179]. Natural pigments are currently used more frequently than that of are chemically synthesized [180]. While red and yellow colorants were once widely employed in food coloring, blue is becoming more and more popular as a food colorant [181]. Polyketide pigments of *Monascus*, which produce a variety of red, yellow, orange, green, and blue hues, have great potential in this regard [181]. Figure 4 shows the chemical structure of several colorants. The majority of study has focused on the possibility of using fungal pigments in various industries, notably as food colorants or additives in the food industry [113], which has long been known by many researchers [78,152,182].

*Monascus* pigments, Arpink red from *P. oxalicum*, riboflavin from *Ashbya gossypii*, and β-carotene from *B. trispora* have already reached the worldwide market as food colorants (Table 2) [78,183]. These fungal pigments also have good commercial production yields. For example, the production yield of β-carotene in a *Blakeslea trispora* culture medium was reported to be 17 g/L [53,184]. In a study by Abdel-Raheam et al. (2022), *Monascus purpureus* was employed as an coloring component in ice lollies. The study found that the ice lolly to which these colors were added was highly accepted [185]. *Monascus* pigments may additionally be applied to other foods, such as fruit-flavored yogurt [186], sweet drops [187], flavored milk [188], jelly beans, and lollipops [189]. *Penicillium brevicompactum* was identified as a novel source of colors for the food sector in a recent study [190].

#### 6.1.1. Application of Anthraquinones

*Penicillium oxalicum* produces the anthraquinone pigment Arpink red, a red pigment with bacteriostatic, antiviral, fungicidal, herbicidal, and insecticidal characteristics [54]. Foodstuffs can be supplemented with the Arpink red polyketide of *Penicillium oxalicum* without any stabilizing [191]. After evaluating the toxicological data of the Arpink red pigment [77], Codex Alimentarius Commmision (CAC) made the statement about the amount to be used in food products (Table 3) that will be non-objectionable [192].

#### 6.1.2. Application of Azaphilones

The chemical structure of azaphilone has been identified in over 50 distinct ways, and it may readily be coupled with nitrogenous compounds [90]. Monascorubrin, an orange azaphilone pigment derived from *Monascus* sp., may combine with amino acids to produce a red hue in meals [175]. Again, the polyketide pigments have improved functionality with respect to light stability, water solubility [193], anti-atherogenic activity [194], and antioxidant properties [195] when added to specific food products. As polyketide pigments, azaphilones (red and yellow colorants) of *Monascus* sp. have been lawfully commercially manufactured and used as food colorants all over the world. In Southeast Asia, a traditionally produced, dry fermented red rice powder has been utilized for over one thousand years [29]. More than 50 patents have recently been issued in several countries, including Japan, the United States, France, and Germany, regarding the use of *Monascus* pigments in food items [174]. It has been shown that several *Talaromyces* species, such as *T. aculeatus*, *T. funiculosus*, *T. pinophilus*, and *T. purpurogenus*, generate azaphilones, *Monascus* pigment analogues (MPA) pigments, similar to those seen in *Monascus* without generating citrinin or any other recognized mycotoxins [95].

#### 6.1.3. Application of Riboflavin

Riboflavin, often known as vitamin B_2_, is a yellow pigment that is used as a food colorant in most countries and is legal to use. Salad, sherbet, drinks, ice creams, pharmaceuticals, and other goods are among the products in which this pigment is utilized [138]. However, because of its slightly unpleasant smell and bitter taste, its use in cereal-based goods is rather limited, despite the fact that it has an affinity for them. Several bacteria create riboflavin through fermentation. Riboflavin can be divided into three types based on fermentation yield: (i) weak overproducers (100 mg/L or less, e.g., *Clostridium acetobutylicum*), (ii) moderate overproducers (600 mg/L or more, e.g., *Candida guilliermundii* or *Debaryomyces subglobosus*), and (iii) strong overproducers (over 1 g/L). Due to the superior genetic stability of its pigment, *Ashbya gossypi* is chosen for fermentation over others [196].

### 6.2. Pigments as Antimicrobial Agents

Fungal pigments, according to several research studies [56], have numerous health benefits over synthetic pigments, including antibacterial action against a variety of harmful bacteria, yeast, and fungi. The researchers also proposed that these bioactive pigments may be employed in the food and pharmaceutical sectors as food preservatives or antibacterial agents [16,183,197]. It has also been studied whether they may be used to make medical items such as bandages, suture threads, and face masks, and the documented findings imply that it is quite possible [198]. The antimicrobial property of the red pigment generated by *M. purpureus* was discovered, and the extract of *M. purpureus* was shown to be 81% effective when compared to the antibiotic ciprofloxacin [199]. Pencolide, sclerotiorin, and isochromophilone were isolated from another fungal strain, *P. sclerotiorum*, in a large-scale liquid culture. Isochromophilone was found to have antibacterial properties against *S. aureus* [200]. It was shown that *Aspergillus sclertiorum* DPUA 585 generated Neoaspergillic acid, which has antibacterial action against *Escherichia coli*, *Mycobacterium smegmatis*, and *Staphylococcus aureus* and antifungal activity against *C. albicans* [60]. Antibacterial activity has also been observed in *Aspergillus versicolor* [61]. Furthermore, antibacterial activity was found in *Penicillium* species isolated from Brazilian cerrado soil, with considerable activity against *C. albicans*, *Listeria monocytogenes*, and *Bacillus cereus*, respectively [201]. A key fungus species in the synthesis of many colors is *Rhodotorula glutinis*. The industrial scale use of this type of yeast has included creating carotenoid colors and acting as a biological control against the post-harvest degradation of fruit [202]. *Rhodotorula glutinis* pigment may effectively kill both the planktonic type of food-spoilage bacteria and the bacteria that form food-spoilage biofilms [203]. *Aspergillus nidulans* JAS3, an Indian-Ocean-isolated pigmented fungal strain, was recently the subject of a study that included its extraction, characterization, and antagonistic activity toward clinical pathogens. When strain JAS3 was treated in enhanced Czapek Dox medium at 28 °C, it was discovered that the pigment it produced was of a pale yellow hue. When tested against several clinical pathogenic strains, the colored pigment demonstrated good bioactivity, including antimicrobial, anti-proteinase, and antifouling activities [204]. In another study, a pigment derived from *Gonatophrgmium truiniae* was found to have antibacterial properties against *Bacillus subtilis*, *Staphylococcus aureus*, and *Micrococcus luteus* [30]. According to Poorniammal and Prabhu (2022), the fungal pigments produced from *Thermomyces* sp. and *Penicillium purpurogenum* have antibacterial properties that are effective against *Staphylococcus aureus* [205].

### 6.3. Pigments as Antioxidant Agents

Microbial pigments such as carotenoids, violacein, and naphthoquinones have been shown to have antioxidant properties through several studies. The antioxidant potential of pigments from various fungi has been mentioned in a number of review papers [12,177,206]. Studies on the antioxidant activity of pigments from several fungi, including *Penicillium* sp. (*P. miczynskii*, *P. purpureogenum*, *P. purpuroscens*), *Fusarium* sp., *Thermomyces* sp., *Chaetomium* sp., *Sanghuangporus baumii*, *Stemphylium lycopersici*, and *Trichoderma* sp. (*T. afroharzianum*) have revealed their promising antioxidant potential and their possible application in the healthcare industry [207]. *Epicoccum nigrum* has also been demonstrated to be a non-mycotoxigenic fungal producer of a polyketide pigment with antioxidant properties [95]. The extracted pigment generated by *Monascus purpureus* in the investigation. Zeng et al. (2021) showed a stronger antioxidant activity in scavenging free radicals and preventing lipid oxidation [208]. In the study by Nair and Abraham (2023), it was revealed that a pale yellow pigment produced by *Aspergillus nidulans* JAS3 demonstrated antioxidant activity [204]. In another study, *Phoma* sp. RDSE17 was isolated and characterized for its melanin pigment. The biological characteristics of the pure melanin of the fungus were examined for their antioxidant activities. The pure melanin demonstrated strong DPPH free-radical-scavenging activity with an EC_50_ of 69 µg/mL [209]. In the study by Fonseca et al. (2022), natural pigments derived from *Penicillium brevicompactum* were tested and found to be mycotoxin-free with potential antioxidant action [209]. Extracellular fungi pigments from Penicillium murcianum and Talaromyces australis demonstrated biotechnological potential of antioxidant activities in a study [210]. In another study, *Gonatophrgmium truiniae*’s pigment demonstrated antioxidant activity with an IC50 value of 0.99 mg/mL [157].

### 6.4. Pigments as Anticancer Agents

Fungal pigments have been shown to have anticancer and antitumor effects. Several investigations have indicated that fungal pigments might be used as an anticancer medication. Pigments of *Monascus* species (*M. purpureus* and *M. pilosus*) such as monascin, ankaflavin, monaphilone A–B, monapilol A–D, and monapurone A–C have been shown to have anticancer/antitumor potential against various cancers, including mouse skin carcinoma, human laryngeal carcinoma, human colon adenocarcinoma, and human hepatocellular carcinoma [29]. In addition to *Monascus*, other fungal pigments with anticancer, antitumor, or antiproliferative activities include norsolorinic acid from *A. nidulans*, shiraiarin from *Shiraia bambusicola*, alterporriol K, alterporriol L, and alterporriol M from *Alternaria* sp., benzoquinone from *Fusarium* sp., and an uncharacterized red pigment F (MCF-7, MDA-MB-435, and MCF-7 b), whereas hypocrellin D from *S. bambusicola* has anticancer effects against many other cancer cell lines (Bel-7721, A-549, and Anip-973) [67,211]. As an example, the anticancer properties of the AUMC 5705 *Monascus* strain as well as that of the AUMC 4066 secondary metabolites, which have numerous uses in the food, pharmaceutical, and other sectors, are evident [52]. The anticancer potentiality of raw coix seed fermented by *Monascus purpureus* was demonstrated and observed thatthe HEp2 cell line of human laryngeal carcinoma, which makes up 25% of neck and head cancers, was used to test the extract’s anticancer potential [208]. In another study, 80 µg/mL of pure melanin extracted from *Phoma* sp. RDSE17 hindered the development of human lung cancer cells [209].

### 6.5. Pigments Used in Pharmaceuticals

Sclerotiorin, a bioactive metabolite generated by *P. sclerotiorum*, has been utilized in the pharmaceutical sector [82]. *Penicillium* sp. NIMO-02 produces a pigment that is important in the food and pharmaceutical sectors [96]. *P. purpurogenum* generated greater extracellular pigments with antibacterial activity in darkness, which may be used in the pharmaceutical and healthcare industries [212], while *Trichoderma virens* has eco-friendly antifungal characteristics. *Penicillium* sp. generates various secondary metabolites with high bioactive chemicals; these are utilized in pharmacy to make medicines to treat a variety of ailments and in agriculture [213]. *P. oxalicum* var. Armeniaca CCM 8242 generated an anthraquinone chromophore. The anthraquinone derivative Arpink red possesses anticancer properties and is used in food and medicines [6,214]. Sorbicillinoid pigments from Stagonospora sp. SYSU-MS7888 demonstrated anti-inflammatory activity in a recent research study [215]. The effectiveness of a purified anthraquinone from Talaromyces purpureogenus as a powerful agent for kidney radio-imaging, which might be used in the diagnosis of kidney cancer, was demonstrated [215]. As intriguing alternative medication sources, several instances of true endophytic fungi generating anthraquinones similar to their various host plants have been documented [216]. A pale yellow pigment produced by *Aspergillus nidulans* JAS3 was found to have anti-inflammatory activities [204]. According to another study, cadmium can be reduced with melanin pigment derived from *Aspergillus terreus* LCM8 [217].

## 7. Mycotoxins in Fungal Pigments

The study of fungal secondary metabolites would be incomplete without mentioning mycotoxins. Health and productivity can be negatively affected by mycotoxins, which are secondary metabolites generated by many fungi. The most significant variables are carbon and nitrogen supplies, although other factors such as oxygen, metal ions, and temperature also have an impact on polyketide synthesis, including citrinin [218]. Several recognized mycotoxins, such as secalonic acid D, oxaline, citrinin, tanzawaic acid A, cyclochlorotine, islanditoxin, luteoskyrin, erythroskyrin, rugulosin, or aspergiolide A, are co-produced in the medium by *Aspergillus* and *Penicillium* sp. There are commercially available and legally permitted *Monascus* pigments in Japan [219] and Southeast Asia, but they are not allowed in the European Union (EU) and the United States (US) due to the danger of contamination by citrinin, a potentially nephrotoxic and hepatotoxic metabolite [220]. Over the past 20 years, *Monascus* pigment research has focused on strategies to minimize citrinin synthesis or on developing strains that are incapable of co-producing citrinin [221].

Citrinin (also known as monascidin) was discovered in *Monascus* and *Aspergillus* fungal strains after being recognized as a yellow pigment generated by *Penicillium citrinum* [222]. Citrinin has been shown to be genotoxic in vitro and in vivo in several studies. In a mouse study, citrinin caused chromosomal aberrations and breakage in the bone marrow cells of both young weanling and adult mice [223]. In addition, citrinin increased the frequency of micronuclei in human cells in a concentration-dependent manner [224]. The genotoxicity of citrinin is linked to tumorigenicity; after 80 weeks of oral treatment, Fischer 344 rats developed kidney adenomas [225]. Other possible hazardous metabolites, such as monascopyridines [73] and ankaflavin, have exhibited specific cytotoxicity to cancer cell lines via an apoptosis-related mechanism in addition to citrinin. Monascin, on the other hand, has demonstrated no cytotoxicity in any cell lines examined [226]. This suggests that at suitable amounts, both monascin and ankaflavin are harmless, and that ankaflavin may potentially be used as a food colorant.

However, fungal producers are usually classified as generally recognized as safe (GRAS), which means that their mycotoxins can be regulated with constant monitoring. UV radiation and chemical mediators were employed to create low-citrinin-producing mutants, according to one study [16]. Citrinin and additional polyketide biosynthesis genes (pksCT, ctnA, and Mga1, for example) have also been found [227]. Citrinin production can be reduced or eliminated by manipulating culturing conditions [228], developing strains incapable of synthesizing citrinin by metabolic and genetic engineering [229], and simply screening for genera other than *Monascus* that produce polyketide pigments [113]. The discovery of several *Talaromyces* species (*Talaromyces aculeatus*, *T. funiculosum*, *T. pinophilus*, *T. atroroseus*, and *T. albobiverticillius*) that produce *Monascus*-like polyketide azaphilone colors without co-producing citrinin or any other known mycotoxins arose from a thorough search for a suitable strain [95]. Marine fungus is also gaining attention; research has revealed that marine fungi can generate a more vivid color with greater stability and fewer or no mycotoxins.

## 8. Future Prospects

Prior to the beginning of European civilization, when the Aztec, Mesopotamian, and Egyptian societies emerged, humans were infatuated with color. The research and development of microbial pigments, as well as their commercial demand, are increasing [7] as consumers become more aware of the dangers of synthetic colors. The industrial production of natural food colorants has three important areas for the future: the stable production of colorants of a consistent quality, the discovery of new sources of novel or recognized classes of pigments and color hues, and improved usefulness. *Monascus* pigments have long been used to make red rice wine, red soybean cheese, and Anka (red rice) in Southern China, Japan, and Southeast Asia. The mycotoxin citrinin is also produced by the fungus, but there have been no reported cases of death associated with consumption of red rice wine or red soybean cheese. *Penicillium*, in addition to *Monascus*, has been documented for synthesis and subsequent use as a human-friendly pigment.

According to recent research, filamentous fungi might be used as cell factories for pigment synthesis and could be used to change the functioning of natural food colorants and extend the color palette while utilizing fungal diversity. A deeper understanding of how filamentous fungi generate polyketide colors without mycotoxin is, however, definitely needed. Fungal polyketide colorants, similar to other new natural colorants, must be evaluated for toxicity before being approved. Significant work remains to be carried out in exploring fungal biodiversity for biocolors with minimal or zero mycotoxin generation, with a focus on water-soluble pigments. Combinatorial genetic engineering, based on a growing number of known carotenogenic gene sequences, is now being explored. Researchers were able to create more efficient biosynthesis by combining genes or novel carotenoids, including those never documented before, such as multi-hydroxylated carotenoids, which might be highly effective antioxidants.

To fulfil the rising market demand for biopigments, effective extraction and purification procedures, appropriate culture conditions, and better microbial strains must be developed. Another potential method is using agricultural wastes as carbon and nitrogen sources. Given recent developments in this sector, the inclusion of pigment bioproduction into biorefineries may be viable in near future. The demand for biopigments in the food, pharmaceutical, nutraceutical, and textile sectors will be supported by microbial production of biopigments using agro-industrial wastes under a biorefinery platform in the next 5 years. Modern biotechnological interventions in biorefinery will be critical in large-scale biopigment production, ensuring effective commercialization and ultimately boosting the bioeconomy [230] Using agricultural and industrial waste as a raw source can reduce the cost of producing microbial products while also enhancing their sustainability [231].

## 9. Conclusions

As all food additives are subject to stringent regulation and approval, it is critical that the production and purification of microbial pigments do not result in the presence of any undesirable, harmful, or allergenic metabolites in the final product. Natural colors are supposed to be more expensive than their synthetic counterparts. Moreover, several factors, such as stability during harsh physical and chemical processes, the tendency to react with other food compounds, time-consuming extraction and purification processes, the cost of synthetic media for microbial production, and so on are causing the production of natural pigments to be more challenging. Nevertheless, given the numerous advantages of fungal pigments over synthetic pigments, the current societal demand for “natural” ingredients has sparked interest in investigating new methods and sources for biotechnological food colorant production. In this sense, employing proper methods and methodologies and investigating fungal chemical diversity provides a promising path for the identification of new, safe, and eco-friendly pigments. Aside from using a strain that produces high pigment yields, the present and future problems in this field are connected to the safety of end products due to the mycotoxins generated by some fungal strains. More research is needed to ensure enough synthesis and the easy recovery of safe and environmentally acceptable microbial pigments. Furthermore, traditional strain improvement approaches, advanced genetic engineering techniques for strain improvement, genome shuffling, and fermentation strategies to scale up production to industry levels can be applied for the sustainable production of high-use microbial pigments. The colorant genes can be injected into the vector’s genome via the CRISPR-CAS9 system to extrapolate the production of microbial secondary metabolites. Therefore, we may infer that careful observation with or without genetic modification and low-cost technologies, such as the new genome-editing approach CRISPR, can be used to ensure the efficient and regulated synthesis of potentially safe polyketide colors from fungal strains.

## Figures and Tables

**Table 1:** Fungal pigments and their potential applications.

Fungi	Color	Pigment	Molecular Formula	Applications	Status	References
*Ashbya gossypi*	Yellow	Riboflavin	C_17_H_20_N_4_O_6_	Food and beverages	IP	[53]
*Aspergillus awamori* and *Aspergillus niger*	Yellow, Brown	Asperyellone	C_20_H_22_O	Antibacterial activity	RP	[56,57]
*Aspergillus niger*	Black	Aspergillin	C_24_H_35_NO_4_	Antimicrobial activity	RP	[58]
*Aspergillus flavus*	Red	Unknown	Unknown	Antioxidant activity	NK	[59]
*Aspergillus sclerotiorum*	Yellow	Neoaspergillic acid	C_12_H_20_N_2_O_2_	Antibacterial activity	RP	[60]
*Aspergillus versicolor*	Yellow	Asperversin	C_47_H_58_O_10_	Antifungal agent	RP	[61]
*Blakeslea trispora*	Orange to yellow	β-carotene	C_40_H_56_	Food colorant; anticancer and antioxidant activities	IP	[62,63,64]
*Blakeslea trispora*	Red	Lycopene	C_40_H_56_	Food colorant, anticancer	IP	[51]
*Cordyceps unilateralis*	Deep, blood red	Naphtoquinone	C_10_H_6_O_2_	Food colorant; anticancer and antibacterial activities	RP	[63,65,66]
*Fusarium oxyporum*	Pink/violet	Anthraquinone	C_14_H_8_O_2_	Antibacterial activity	IP	[54]
*Fusarium* sp.	Yellow	Benzoquinone	C_6_H_4_O_2_	Anticancer agent		[67]
*Fusarium sporotrichioides*	Yellow to orange/Red	β-carotene/Lycopene	C_40_H_56_	Food colorants	RP	[51,68]
*Fusarium verticillioides*	Yellow	Napthoquinone	C_10_H_6_O_2_	Antibacterial activity and food colorant	RP	[45]
*Lactarius* sp.	Blue	Azulenes	C_10_H_8_	Food and beverages	RP	[53]
*Mucor circinelloides*	Yellow to orange	β-carotene	C_40_H_56_	Food colorant	DS	[69]
*Monascus purpureus* FTC 5357	Red	Monascorubramine	C_28_H_33_NO_8_	Food colorant	RP	[70]
*Monascus ruber* CCT 3802	Orange, yellow, and red	Monascorubrin	C_23_H_26_O_5_	Food colorant	RP	[71]
*Monascus purpureus*	Yellow	Monascin	C_21_H_26_O_5_	Food colorant	IP	[72]
*Monascus* sp.	Yellow	Ankaflavin (Azaphilone)	C_23_H_30_O_5_	Food colorant, pharmaceutical, and antitumor and antiinflamatory activities	IP	[51,63]
*Monascus* sp.	Orange	Rubropuntatin	C_21_H_22_O_5_	Anticancer activity and food colorant	IP	[1,52,67]
*Monascus* sp.	Red	Monascorubramine	C_23_H_27_O_4_	Antioxidant activity and food colorant	IP	[29,50,51]
*Monascus* sp.	Red	Monascopyridine B	C_23_H_29_NO_4_	Antioxidant activity	IP	[73]
*Monascus roseus*	Orange, red/pink	Canthaxanthin	C_40_H_52_O_2_	Antioxidant and anticancer activities	IP	[51,74]
*Neurospora crassa*	Yellow to orange	β-carotene	C_40_H_56_	Food colorant	RP	[75]
*Paecilomyces sinclairii*	Red	Unknown	---------	Food colorant	RP	[51]
*Penicillium herquei*	Yellow/blue	Atrovenetin	C_19_H_17_O_6_	Antioxidant and food colorant		[76]
*Penicillium oxalicum*	Arpink red and other hue	Anthraquinone	C_14_H_8_O_2_	Anticancer activity in food and pharmaceuticals; antifungal and virucidal activities	IP	[15,49,67,77,78]
*Penicillium purpurogenum*	Orange/Yellow to orange/Red/orange to red	Purpurogenone/Mitorubrin/Azaphilone/Mitorubrinol	C_14_H_12_O_5_/C_21_H_18_O_7_/C_21_H_22_O_7_/C_21_H_18_O_8_	Food, antioxidant, and pharmaceuticals	DS	[63,79,80,81]
*Phycomyces* *Blakesleeanus*	Yellow to orange	β-carotene	C_40_H_56_	----------	RP	[62]
*Penicillium sclerotiorum*	Yellow to orange	Sclerotiorin	C_21_H_23_ClO_5_	Antibacterial and antifungal activities	NK	[82]
*Phycomyces blakesleeanus*	Yellow to orange	β-carotene	C_40_H_56_	Food colorant	RP	[83]
*Pseudoalteromonas* *Denitrificans*	Red	Cycloprodigiosin		Antiplasmoidal and anticancer activities	DS	[84]
*Stemphylium lycopersici*	Red	Anthraquinone	C_14_H_8_O_2_	Antioxidant activity		[85]
*Talaromyces atroroseus*	Red	Azaphilone	C_21_H_22_O_7_	Food colorant and antioxidant and anticancer activities	DS	[14,51]
*Talaromyces* sp.	Red	N-glutarylmonascorubramine	C_28_H_33_NO_8_	Food colorant	IP	[86]
*Trichoderma virens*	Yellow	Virone	C_22_H_24_O_4_	Antifungal activity	NK	[11,87]

DS—development stage; IP—industrial production; RP—research project; NK—not known.

**Table 2:** Some authorized food-grade fungal pigments available in the current global market [74,190].

Color	E-Number *	Fungal Pigments	Responsible Fungi
Yellow	E101 (iii)	Riboflavin	*Ashbya gossypii*
Orange-yellow	E160a (ii)	Β-carotene	*Blakesla trispora*
Yellow to red	E160d (iii)	Lycopene	*Blakesla trispora*
Yellow/orange/red	E161g	Canthaxanthin	-------------

* E-number represents the corresponding authorized food colorants in the European Union.

**Table 3:** Use of anthraquinone (Arpink red) pigment in various food products.

Sample (Food Products)	Anthraquinone (Amount mg/kg)
Milk products	150
Ice cream	150
Meat and meat products	100
Nonalcoholic drinks	100
Alcoholic drinks	200
Confectionary products	300

## Data Availability

No applicable.
